# The multidimensional effects of sleep quality in young adults: the relationship between auditory performance, sound tolerance, and anxiety

**DOI:** 10.1007/s00405-025-09979-5

**Published:** 2026-01-21

**Authors:** Aysegul Esdogan, Beyza Demirtaş Yılmaz

**Affiliations:** 1 Department of Audiology, Faculty of Health Sciences, KTO Karatay University, Konya, Turkey; 2 Department of Audiology, Faculty of Health Sciences, Erciyes University, Kayseri, Turkey

**Keywords:** Sleep quality, Auditory system, Hyperacusis, Misophonia

## Abstract

**Purpose:**

This study aimed to comprehensively evaluate the effects of sleep quality on auditory performance, sound tolerance, and anxiety levels in young adults and to examine the possible relationships between these variables.

**Materials and methods:**

The study included 607 young adults aged 18-30 years (mean 21.48 ± 2.88 years). Participants completed a participant information form created for this study, the Pittsburgh Sleep Quality Index (PSQI), the Decreased Sound Tolerance Scale-Screening (DSTS-S), the Speech, Spatial, and Hearing Qualities Scale (SSQ), and the Beck Anxiety Scale. Data analysis was performed using IBM SPSS Statistics (Version 23.0) software.

**Results:**

The participants’ PSQI mean was 7.62±3.76, and 65.53% of the participants (>5) showed poor sleep quality. PSQI scores showed a negative correlation with SSQ total scores (rₛ=-0.242, *p*<0.001) and a moderate positive correlation with Beck Anxiety scores (rₛ=0.420, *p*<0.001). Furthermore, PSQI showed significant positive correlations with hyperacusis (rₛ=0.294, *p*<0.001), phonophobia (rₛ=0.159, *p*=0.001), and misophonia (rₛ=0.312, *p*<0.001).

**Conclusion:**

This study revealed statistically significant relationships between poor sleep quality in young adults and auditory performance in daily life, reduced sound tolerance, and anxiety levels. The findings of the present study indicate that poor sleep quality is significantly associated with lower auditory performance and sound tolerance. Furthermore, poor sleep quality was found to be associated with higher anxiety levels. These findings demonstrate the effects of sleep on various systems and emphasize its physiological necessity for healthy functioning.

## Introduction

Sleep is one of the fundamental operational states of the central nervous system and an important psychophysiological process for brain function and mental health [1]. Sleep quality is a complex parameter that encompasses subjective sleep perception and sleep duration, continuity, structure, and restorative value [2]. Poor sleep quality has been reported to be exceptionally prominent among young adults [3, 4]. In recent years, screen time, addiction to smartphones, caffeine consumption, eating habits, and physical activity levels in young adults have been shown to have an impact on sleep quality [5–7]. It has been theorized that the degradation of sleep quality associated with changes in diet and lifestyle patterns can also have a negative impact on physiological body processes [8, 9]. Sleep problems are more common in the young adult population, and it is stated that these individuals are more affected and more vulnerable to sleep deprivation than older adults [4, 10].

Sleep disorders can impair cochlear function by causing physiological changes such as a decrease in oxygen saturation, which can lead to adverse effects on the auditory system [11, 12]. However, studies examining the relationship between sleep quality and auditory skills are quite limited. For example, Ren et al. [13] observed a significant increase in P300 latency and reaction time in participants after 24 h of sleep deprivation. Furthermore, Hébert et al. [14] reported a significant relationship between sleep complaints and symptoms of hyperacusis, a condition characterized by reduced sound tolerance.

One of the basic functions of sleep is to maintain synaptic homeostasis [15]. It has been reported that sleep deprivation has adverse effects on cognitive functions and mood due to the disruption of synaptic homeostasis between cells [4, 16]. The literature describes a bidirectional relationship in which anxiety disorders may be a risk factor for sleep disorders, and sleep disorders may be a risk factor for anxiety disorders [15, 17]. However, although sleep complaints are frequently seen in individuals with anxiety disorders, polysomnographic findings show no significant difference in sleep onset latency and sleep continuity. It has been suggested that the inconsistencies in the findings of the studies may be due to the presence of various factors that can affect sleep quality, as anxiety is associated with many psychiatric disorders [18].

Current findings suggest that sleep quality can have a significant impact on sensory systems. In particular, the potential relationship between sleep quality and the auditory system is an area of research that has yet to be fully elucidated. Preliminary findings suggest that sleep disorders may impact an individual’s auditory performance and sound tolerance, underscoring the need for more comprehensive studies on this topic. Furthermore, while it is known that sleep disorders can negatively affect an individual’s mental health and may be a risk factor for anxiety disorders, it is important to elucidate this relationship fully.

The aim of the current study was to comprehensively assess the effects of self-reported sleep quality in young adults on auditory performance, sound tolerance, and anxiety levels and to examine potential relationships between these variables. Considering that young adults’ sleep patterns are more easily disrupted due to their lifestyle and they are more vulnerable to sleep disorders compared to older adults, this age group was chosen as an appropriate study group to examine the multidimensional effects of sleep. Based on the existing literature, it is hypothesized that lower sleep quality will be associated with lower auditory performance and reduced sound tolerance. However, higher anxiety levels are expected to be associated with both poor sleep quality and impaired auditory outcomes. Finally, it is predicted that sleep quality will be an important predictor of auditory performance and sound tolerance, and that anxiety may potentially mediate these relationships.

## Materials and methods

### Ethical approval

This study is a cross-sectional research, and ethical approval was obtained from the KTO Karatay University Faculty of Medicine Ethics Committee for Non-Drug and Non-Medical Device Research with decision number 2025/026. The study was conducted in accordance with the principles outlined in the Declaration of Helsinki.

### Participants

The inclusion criteria for the study were: being between 18 and 30 years of age, having normal hearing, having no history of metabolic, neurological, psychological, or psychiatric disorders, and having no diagnosed sleep disorder.

### Data collection

The study data were collected between January and May 2025 by two independent audiologists in separate sessions. Participants were approached to engage in the study through direct face-to-face interviews and informed about the study’s aim. Voluntary participation was obtained, and written consent was achieved for all participants. Data collection was conducted in two sessions. Participants completed the Pittsburgh Sleep Quality Index (PSQI), the Decreased Sound Tolerance Scale-Screening (DSTS-S), and a participant form developed for this study at the first session. They filled out the Beck Anxiety Inventory and the Speech, Spatial and Qualities of Hearing Scale (SSQ) at the second session. Data collection took an average of 40 min per interviewee, with a 15-minute pause between sessions to allow respondents to rest and ensure that the responses were completed reliably.

#### Data collection tools

Data on the participants’ characteristics and health status, such as age, gender, smoking status, having chronic diseases, psychiatric diagnosis history, migraine, sleep apnea, hearing loss, and ear health, were gathered via a personal information form. The participants’ lifestyle behaviors on a day-to-day basis were also monitored, for example, the average time spent in front of a screen, how often they were exposed to loud places, how many hours they listen to music, and how much caffeine they consume.

##### Pittsburgh sleep quality index (PSQI)

Buysse et al. [2] conceptualized the PSQI, a 19-item self-report measure assessing the quality of sleep and sleep disturbances over the past month. Seven components of this scale include subjective sleep quality, sleep latency, duration, usual sleep efficiency, sleep disruptions, sleep medication use, and daytime functioning. Ağargün et al. [19] performed a validity and reliability study of the scale on Turkish populations. The total score was calculated using 18 items, each scoring between 0 and 3 on the scale. The total PSQI score ranges from 0 to 21, representing the sum of the scores for the seven components included in the scale. A total PSQI score of ≤5 indicates ‘good sleep quality’, while a score >5 indicates ‘poor sleep quality’. The Cronbach’s alpha internal consistency coefficient of the scale was obtained as 0.80.

##### DSTS-S (Decreased sound tolerance scale-screening)

Alluşoğlu and Aksoy [20] created the Decreased Sound Tolerance Scale-Screening (DSTS-S) as a screening tool for differentiating between the three subtypes of decreased sound tolerance: misophonia, phonophobia, and hyperacusis. It is a 31-item scale measuring misophonia (14 items), phonophobia (5 items), and hyperacusis (12 items). It uses a Likert scale of 0 to 3 (0: Never, 3: Always) to rate each item. The misophonia, phonophobia, and hyperacusis sections of the scale have Cronbach’s alpha coefficients of 0.881, 0.775, and 0.938, respectively.

##### SSQ (Speech, spatial, and qualities of hearing scale)

William Noble and Stuart Gatehouse created the Speech, Spatial, and Qualities of Hearing Scale (SSQ) in 2004 to evaluate various types of adult hearing impairment [21]. The 49-item Turkish version, translated by Kılıç et al. [22], is represented in three subscales: “Speech Perception” (14 items), “Spatial Perception” (17 items), and “Hearing Quality” (18 items). Each of the 49 items also has scores ranging from 0 to 10 (0:definitely not, 10: perfectly). By adding up all of the item scores and dividing by the number of items (49), the average SSQ score is determined. The total score for each subscale is obtained by dividing the total score by the number of questions in the relevant subscale. The Cronbach’s alpha value of the TR-SSQ scale was found to be 0.984.

##### Beck anxiety inventory

This scale, developed by Beck et al. [23] measures the severity of anxiety. A Turkish validity and reliability study of the scale was conducted by Ulusoy et al. [24]. It is a 21-item self-report scale. Each item is scored from 0 to 3, and the total score obtained from the scale ranges from 0 to 63. The total score obtained from the scale is interpreted as follows: 0-7 indicates minimal anxiety, 8-15 indicates mild anxiety, 16-25 indicates moderate anxiety, and 26-63 indicates severe anxiety [23]. The Cronbach’s alpha value of the Turkish version of the Beck Anxiety Scale was found to be 0.93 [24].

### Data analysis

Statistical analyses were performed using IBM SPSS Statistics (Version 23.0, IBM Corp., Armonk, NY, USA) software. Descriptive statistics for continuous variables are expressed as mean ± standard deviation (Mean ± SD) and median [interquartile range] (Median [IQR]), while categorical variables are presented as frequency (n) and percentage (%).In our study, data normality was tested using the Shapiro-Wilk test, and nonparametric tests were preferred because variables such as the PSQI, Beck Anxiety, and DSTS-S tests did not show normality.Differences between two independent groups (gender, presence of tinnitus) were analyzed using the Mann-Whitney U test and the Kruskal-Wallis H test was used to compare more than two independent groups (music listening time, caffeine consumption, and screen time). In cases where the Kruskal-Wallis H test results were significant, post-hoc pairwise comparisons were made using the Mann-Whitney U test with Bonferroni correction. Effect sizes for group comparisons were calculated using the formulas r = Z/√N for the Mann-Whitney U test and ε² = χ²/(N-1) for the Kruskal-Wallis test. Spearman’s Rho (rₛ) correlation coefficient was used to assess the relationships between variables. A Bonferroni correction was applied to reduce the risk of Type I error arising from multiple comparisons, and adjusted p values ​​(p Bonf.) are reported. A series of multiple linear regression analyses were performed to investigate potential predictors of the primary dependent variables (PSQI, sound tolerance scales, SSQ subscales, and total score). All independent variables (Beck Anxiety score, presence of gender-related tinnitus, duration of music listening, caffeine consumption, and screen time) were simultaneously included in the regression model, and model fit was assessed using the adjusted R² value. A significance level of *p* < 0.05 was accepted for all statistical tests.

## Results

A total of 607 individuals were included in the study, with an average age of 21.48 ± 2.88 years. 74.1% of participants were female (*n*=450) and 25.9% were male (*n*=157). In the current study, tinnitus symptoms were present in 12.2% of participants (*n* = 74), while 87.8% (*n* = 533) reported no symptoms. In terms of anxiety level, mild anxiety was detected in 28.2% of participants (*n*=171), moderate anxiety in 34.1% (*n*=207), and severe anxiety in 31.30% (*n*=190). Most participants (74.6%) used screens for more than 3 h per day, and 44.6% listened to music for 1–3 h daily. In terms of caffeine consumption, 41.8% of participants (*n*=254) consumed caffeine frequently (See Table 1).

**Table 1:** Descriptive statistics

Variable	Category	*n*	%
Gender	Male	157	25.9
	Female	450	74.1
Tinnitus	No	533	87.8
	Yes	74	12.2
Anxiety level	None	39	6.4
	Mild	171	28.2
	Moderate	207	34.1
	Severe	190	31.3
Screen time	<1 h	20	3.3
	1–3 h	134	22.1
	>3 h	453	74.6
Music duration	<1 h	199	32.8
	1–3 h	271	44.6
	>3 h	137	22.6
Caffeine use	Rarely	123	20.3
	Sometimes	230	37.9
	Frequently	254	41.8

Table 2 presents the mean, standard deviation, and score ranges for the PSQI, Beck Anxiety, DSTS-S (hyperacusis, phonophobia, misophonia), SSQ subscales, and SSQ total score. The mean PSQI score for the individuals in the study was 7.62±3.76, with 68.53% of participants exceeding the threshold for poor sleep quality (PSQI>5). Beck Anxiety scores (21.00 ± 13.83) showed a wide distribution among individuals. When examining the DSTS-S subscales, hyperacusis and misophonia scores were found to be higher, while phonophobia scores were relatively lower. SSQ subscales and total scores were generally found to be at a good level, but significant individual differences were observed.

**Table 2:** Descriptive statistics of the scales

Scale	Mean ± SD	Min–Max
PSQI	7.62 ± 3.76	0–21
Beck Anxiety Inventory	21.00 ± 13.83	0–60
DSTS-S Hyperacusis	10.86 ± 6.38	0–36
DSTS-S Phonophobia	2.62 ± 2.87	0–15
DSTS-S Misophonia	15.21 ± 11.45	0–42
SSQ Speech Perception	6.99 ± 1.74	2–15
SSQ Spatial Perception	7.27 ± 1.98	3–38
SSQ Hearing Quality	7.40 ± 1.54	3–13
SSQ (Overall Score)	7.24 ± 1.48	3–18

In intergroup comparisons, PSQI questionnaire scores were compared according to gender (U=29982.0, Z= −2.824, *p*=0.018), presence of tinnitus (U=13318.0, Z= −4.530, *p*<0.001), music listening duration (χ²=15.72, *p*=0.002), and caffeine consumption level (χ²=11.12, *p*=0.015) revealing significant differences. Similarly, Beck Anxiety Inventory scores showed significant differences according to gender (U=26880.0, Z=−4.464, *p*<0.001), presence of tinnitus (U=13822.0, Z=−4.173, *p*<0.001), and music duration (χ²=22.34, *p*<0.001) (See Table 3).

**Table 3:** Effects of various factors on PSQI, beck anxiety

Scale	Group	Category	Median (IQR)	*p* (Bonf.)	*r*
PSQI	Gender	Female	7.00 (5.00–10.00)	0.018*	0.115
		Male	6.00 (4.00–9.00)		
	Tinnitus	No	7.00 (5.00–10.00)	0.000***	0.184
		Yes	9.00 (7.00–11.00)		
	Music duration	<1 h	7.00 (5.00–10.00)	0.002**	0.023
		1–3 h	7.00 (5.00–9.00)		
		≥3 h	8.00 (6.00–12.00)		
	Caffeine	Rarely	7.00 (4.00–9.00)	0.015*	0.015
		Sometimes	7.00 (5.00–10.00)		
		Frequently	8.00 (6.00–11.00)		
Beck Anxiety	Gender	Female	15.00 (8.00–23.00)	0.000***	0.181
		Male	21.00 (12.00–33.00)		
	Tinnitus	No	19.00 (10.00–30.00)	0.000***	0.169
		Yes	29.00 (17.25–37.00)		
	Music duration	<1 h	17.00 (8.00–26.50)	0.000***	0.034
		1–3 h	19.00 (10.00–29.00)		
		≥3 h	25.00 (14.00–36.00)		
	Caffeine	Rarely	20.00 (9.50–31.00)	0.645	0.003
		Sometimes	18.00 (10.00–29.00)		
		Frequently	21.00 (12.00–32.00)		

In the SSQ questionnaire, the speech (U=24123.0, Z=3.114, *p*=0.007), spatial (U=23360.0, Z=2.575, *p*=0.040), and auditory quality (U=24303.0, Z=3.241, *p*=0.005) subscales were lower in participants with tinnitus. Additionally, significant differences were observed in the speech (U=40778.0, Z=2.882, *p*=0.016) and spatial (U=40642.0, Z=2.810, *p*=0.020)subscales based on gender. The SSQ questionnaire total scores were also found to be significantly lower in participants with tinnitus (U=24311.0, Z=3.247, *p*=0.005) (See Table 4).

Hyperacusis scores were significantly higher in relation to the presence of tinnitus (U=24857.0, Z=3.633, *p*=0.001). Furthermore, a significant difference was observed in Hyperacusis scores based on music listening duration (χ²=9.119, *p*=0.042). Misophonia scores showed significant differences according to both gender (U=40375.0, Z=2.669, *p*=0.030) and the presence of tinnitus (U=23747.5, Z=2.848, *p*=0.017). Additionally, Phonophobia scores did not show significant differences based on the presence of tinnitus (U=23092.5, Z=2.385, *p*=0.061) and music listening duration (χ²=6.076, *p*=0.192) (See Table 4).

**Table 4:** Effects of various factors on DSTS and SSQ

Scale	Group	Category	Median (IQR)	*p* (Bonf.)	*r*
DSTS Hyperacusis	Gender	Female	10.00 (6.00–15.00)	0.865	0.050
		Male	9.00 (6.00–14.00)		
	Tinnitus	No	10.00 (6.00–14.00)	0.001**	0.147
		Yes	13.00 (8.00–18.00)		
	Music duration	<1 h	11.00 (7.00–15.50)	0.042*	−0.056
		1–3 h	9.00 (6.00–14.00)		
		≥3 h	10.00 (6.00–15.00)		
	Caffeine	Rarely	10.00 (6.00–15.00)	1.000	−0.006
		Sometimes	10.00 (6.00–13.00)		
		Frequently	10.00 (6.00–16.00)		
DSTS Phonophobia	Gender	Female	2.00 (0.00–4.00)	1.000	0.020
		Male	2.00 (0.00–5.00)		
	Tinnitus	No	2.00 (0.00–4.00)	0.061	0.097
		Yes	2.50 (1.00–5.00)		
	Music duration	<1 h	2.00 (0.00–5.00)	0.192	0.012
		1–3 h	2.00 (0.00–3.50)		
		≥3 h	2.00 (1.00–4.00)		
	Caffeine	Rarely	2.00 (0.00–5.00.00.00)	1.000	−0.023
		Sometimes	2.00 (0.00–4.00.00.00)		
		Frequently	2.00 (0.00–4.00.00.00)		
DSTS Misophonia	Gender	Female	14.00 (7.00–23.00)	0.030*	0.108
		Male	13.00 (4.00–19.00)		
	Tinnitus	No	14.00 (6.00–22.00)	0.017*	0.116
		Yes	16.50 (12.00–25.00)		
	Music duration	<1 h	14.00 (7.00–22.00)	0.222	0.036
		1–3 h	13.00 (5.50–20.00)		
		≥3 h	16.00 (7.00–27.00)		
	Caffeine	Rarely	13.00 (6.00–20.50.00.50)	1.000	0.057
		Sometimes	14.00 (7.00–21.00.00.00)		
		Frequently	14.00 (6.00–26.75.00.75)		
SSQ Speech	Gender	Female	7.50 (5.93–8.71)	0.016*	0.117
		Male	7.00 (5.64–8.14)		
	Tinnitus	No	7.14 (5.93–8.29)	0.007**	−0.126
		Yes	6.33 (5.21–7.55)		
	Music duration	<1 h	7.07 (5.79–8.29)	1.000	−0.025
		1–3 h	7.21 (5.86–8.32)		
		≥3 h	6.93 (5.64–8.07)		
	Caffeine	Rarely	7.00 (5.61–8.50)	1.000	−0.006
		Sometimes	7.25 (5.73–8.29)		
		Frequently	7.00 (5.93–8.14)		
SSQ Spatial	Gender	Female	7.53 (6.50–8.59)	0.020*	0.114
		Male	7.12 (6.00–8.24)		
	Tinnitus	No	7.29 (6.18–8.35)	0.040*	−0.104
		Yes	6.65 (5.62–7.80)		
	Music duration	<1 h	7.41 (6.12–8.35)	1.000	−0.030
		1–3 h	7.12 (6.12–8.24)		
		≥3 h	7.12 (6.00–8.41)		
	Caffeine	Rarely	7.00 (6.00–8.29)	1.000	0.007
		Sometimes	7.29 (6.13–8.41)		
		Frequently	7.24 (6.12–8.24)		
SSQ Quality	Gender	Female	7.44 (6.33–8.39)	1.000	0.014
		Male	7.56 (6.44–8.56)		
	Tinnitus	No	7.61 (6.56–8.56)	0.005**	−0.132
		Yes	7.03 (5.47–7.94)		
	Music duration	<1 h	7.56 (6.50–8.78)	1.000	−0.051
		1–3 h	7.56 (6.56–8.50)		
		≥3 h	7.39 (6.11–8.28)		
	Caffeine	Rarely	7.50 (5.95–8.72)	1.000	0.051
		Sometimes	7.47 (6.33–8.32)		
		Frequently	7.70 (6.61–8.61)		
SSQ Total	Gender	Female	7.39 (6.39–8.63)	0.247	0.076
		Male	7.24 (6.18–8.22)		
	Tinnitus	No	7.35 (6.27–8.37)	0.005**	0.132
		Yes	6.73 (5.67–7.66)		
	Music duration	<1 h	7.41 (6.25–8.33)	1.000	0.036
		1–3 h	7.31 (6.20–8.33)		
		≥3 h	7.06 (6.14–8.14)		
	Caffeine	Rarely	7.22 (5.83–8.36)	1.000	0.021
		Sometimes	7.36 (6.20–8.30)		
		Frequently	7.30 (6.27–8.33)		

*Data are presented as median (IQR). Mann-Whitney U and Kruskal-Wallis tests were used. r: Effect size. *: *p* < 0.05, **: *p* < 0.01, ***: *p* < 0.001

**Fig. 1:**
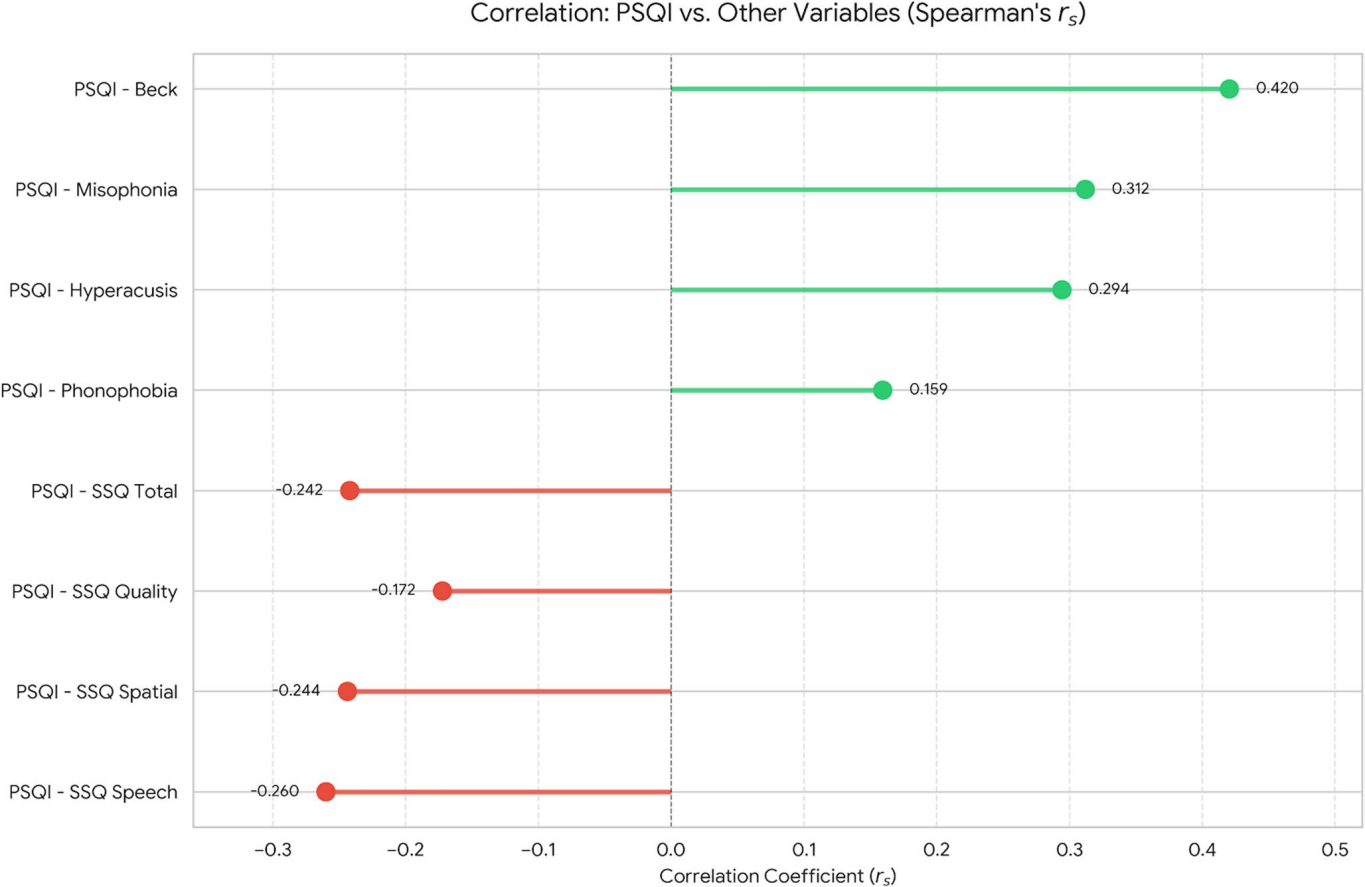
Correlations between PSQI, SSQ, beck anxiety inventory, and DSTS-S (Hyperacusis, Phonophobia, and Misophonia subscales)

The analysis revealed significant correlations between poor sleep quality (higher PSQI scores) and all clinical variables. Specifically, PSQI was moderately positively correlated with Beck Anxiety (rs = 0.420, *p* < 0.001). Regarding sound tolerance, PSQI showed positive correlations with Misophonia (rs = 0.312, *p* < 0.001), Hyperacusis (rs = 0.294, *p* < 0.001), and a weaker but significant correlation with Phonophobia (rs = 0.159, p **=** 0.001). Furthermore, a negative correlation was observed between PSQI and auditory self-perception, as indicated by the SSQ Total score (rs = −0.242, *p* < 0.001) (Fig. 1).

Multivariable linear regression analyses are summarized in Table 5. Across all models, Beck Anxiety emerged as the most consistent and strongest predictor. Higher anxiety scores were independently associated with poorer sleep quality (PSQI), increased sound intolerance (Hyperacusis, Misophonia, and Phonophobia), and lower auditory self-report performance (SSQ Total and all subscales) (all *p* < 0.001).Although univariate analyses showed gender-related differences, gender (female vs. male) was not a significant predictor in any multivariable model after adjusting for anxiety and presence of tinnitus (*p* > 0.05). These findings indicate that interindividual variability in sleep and auditory outcomes was more strongly related to anxiety levels than to gender.Tinnitus remained an independent predictor for PSQI, Hyperacusis, SSQ Quality of Hearing, and SSQ Total. Regarding environmental covariates, frequent caffeine consumption was associated with poorer sleep quality (PSQI; *p* = 0.018). Screen time ≥3 h per day was associated with higher Phonophobia scores (*p* < 0.001) and with lower SSQ Quality (*p* = 0.009) and SSQ Total (*p* = 0.046). Additionally, music exposure of 1–3 h per day was independently related to increased Hyperacusis scores (*p* = 0.005). Unlike the robust and consistent pattern observed for anxiety, these lifestyle variables did not appear as common predictors across all sleep and auditory measures.

**Table 5:** Predictors of PSQI, SSQ, and DSTS-S subscales (Hyperacusis, Misophonia, Phonophobia)

Dependent variable	Predictors	B	*p*	95% CI for B	Adj. *R*²
PSQI (Sleep Quality)	Gender (Female)	0.29	0.380	−0.35 – 0.93	197
	Beck Anxiety Score	0.10	<0.001	0.08–0.12	
	Tinnitus (Yes)	1.20	0.005	0.37–2.04	
	Caffeine (Frequently)	0.90	0.018	0.15–1.64	
Hyperacusis	Gender (Female)	−0.19	0.737	−1.31 – 0.93	123
	Beck Anxiety Score	0.15	<0.001	0.11–0.18	
	Tinnitus (Yes)	1.71	0.024	0.23–3.20	
	Music (1–3 h)	−1.59	0.005	−2.70 – −0.49	
Phonophobia	Gender (Female)	−0.28	0.307	−0.81 – 0.25	73
	Beck Anxiety Score	0.04	<0.001	0.03–0.06	
	Tinnitus (Yes)	0.46	0.187	−0.23 – 1.15	
	Screen (≥3 h)	−2.51	<0.001	−3.77 – −1.25	
Misophonia	Gender (Female)	1.54	0.117	−0.39 – 3.46	103
	Beck Anxiety Score	0.26	<0.001	0.20–0.33	
	Tinnitus (Yes)	1.65	0.300	−1.27 – 4.12	
SSQ Speech	Gender (Female)	−0.27	0.089	−0.59 – 0.04	85
	Beck Anxiety Score	−0.04	<0.001	−0.05 – −0.03	
	Tinnitus (Yes)	−0.29	0.169	−0.70 – 0.12	
SSQ Spatial	Gender (Female)	−0.16	0.382	−0.52 – 0.20	40
	Beck Anxiety Score	−0.03	<0.001	−0.04 – −0.02	
	Tinnitus (Yes)	−0.36	0.141	−0.85 – 0.12	
SSQ Quality	Gender (Female)	0.11	0.446	−0.17 – 0.39	51
	Beck Anxiety Score	−0.02	<0.001	−0.03 – −0.01	
	Tinnitus (Yes)	−0.54	0.004	−0.91 – −0.17	
	Screen (≥3 h)	0.91	0.009	0.23–1.59	
SSQ Total	Gender (Female)	−0.09	0.501	−0.37 – 0.18	73
	Beck Anxiety Score	−0.03	<0.001	−0.04 – −0.02	
	Tinnitus (Yes)	−0.40	0.024	−0.76 – −0.05	
	Screen (≥3 h)	0.66	0.046	0.01–1.31	

Multivariable linear regression analysis was used. Abbreviations: *B* Unstandardized regression coefficient, *CI* Confidence interval, *Adj. R²* Adjusted R-squared, *PSQI* Pittsburgh Sleep Quality Index, *SSQ* Speech, Spatial and Qualities of Hearing Scale. Statistical significance was set at *p* < 0.05$

## Discussion

Sleep is a fundamental factor in maintaining physical and emotional health, and it serves multiple functions [25, 26]. It is recommended that adults get at least 7 h of sleep every night to support optimal health. Consistently sleeping less than 7 h has been associated with adverse health outcomes such as obesity, hypertension, diabetes, heart disease, depression, and increased risk of death [27]. Although healthy sleep is a vital necessity, sleep problems are a growing public health issue affecting 20–45% of the world’s population [26]. It has been noted that changing sleep patterns, particularly among young adults, due to various factors (prolonged technology use, dietary habits, etc.), lead to poor sleep quality [5–7].

Shintya & Wasir [28] reported that the majority of young adults, including university students, experienced poor sleep quality, and that there was a significant relationship between sleep patterns and both coffee consumption and screen time. Similarly, in the current study, a large proportion of young adult participants (68.5%) had poor sleep quality, and their PSQI scores showed significant differences according to caffeine consumption levels. Furthermore, Gardiner et al. [29] reported in their study that higher doses of caffeine consumption and caffeine use later in the evening had a greater negative effect on sleep quality. Adenosine is a receptor that plays an important role in sleep homeostasis by inhibiting cholinergic neurons in the basal forebrain, which promote arousal. Caffeine, on the other hand, primarily supports wakefulness and delays sleep onset by blocking adenosine receptors. This mechanism explains the effect of caffeine consumption (depending on its frequency and timing) on sleep quality deterioration [30, 31].

According to Bhatt et al. [32] tinnitus can be a sign and it can cause anxiety, depression, and sleeping disorders. According to earlier research, individuals with tinnitus tend to experience poor sleep quality [33, 34]. The participants in the current study who reported tinnitus reported statistically significantly poorer quality of sleep compared to those without tinnitus. Insomnia may be triggered by psychological disorders like anxiety and stress caused by tinnitus, while sleep disturbance might result from physiological hyperstimulation in tinnitus patients [35].

Maintaining a regular and adequate flow of oxygen to the cochlea is critical for the healthy functioning of the inner ear. Any decrease in this flow can reduce cochlear sensitivity [36]. Impaired sleep quality can affect the circulatory system, reducing the amount of oxygen reaching the cochlea; this can have long-term adverse effects on the auditory system [37]. By following participants for an average of four years, a study on the UK Biobank cohort investigated the connection between hearing loss and sleep duration and quality. According to this study, there was no significant correlation between the length of sleep and the risk of hearing loss; however, participants’ reported risk of hearing loss increased as their sleep quality declined [38]. Jiang et al. [39] found that excessive daytime sleepiness reported in late middle age was associated with hearing loss 20 years later in older age. Furthermore, it was noted that longer sleep duration in individuals who slept more than 8 h per day during this period reduced their performance in understanding speech in noise in older age. Based on the research findings, sleep disorders can potentially increase the risk of hearing loss in advanced age, and various aspects of sleep can impact peripheral and central auditory function differently. The SSQ scale was used in this study to evaluate the impact of sleep quality on auditory function subjectively. By estimating complex auditory skills, such as spatial hearing, competitive speech perception, and sound discrimination this scale quantifies the daily experience of hearing [21]. The present study found a negative relationship between the sum of PSQI and SSQ scores.These findings, derived from self-report measures, suggest that deteriorating sleep quality exerts a negative impact on auditory functioning in daily life. This effect may be due to sleep deprivation impacting prefrontal cortex functions related to attention and executive processes [40] and reducing neural circuit efficiency by disrupting synaptic homeostasis [41]. As measured by the SSQ, this mechanism provides a physiological explanation for why individuals with poor sleep quality tend to perform worse in challenging listening conditions.

Decreased Sound Tolerance (DST) is a negative reaction to sounds that do not normally cause any discomfort and is defined in 3 subclasses as hyperacusis, misophonia and phonophobia [20]. Hébert & Carrier [14] indicated that mean PSQI scores of old tinnitus patients were related to hyperacusis, citing sleep complaints of this group significantly due to the symptoms of hyperacusis. Similarly, in our study sleep quality showed a significant positive correlation with hyperacusis, phonophobia and misophonia. This suggests that high-intensity phonophobia, misophonia, and hyperacusis symptoms can reduce the quality of sleep. At the neurological level, it is known that sleep deprivation reduces sensory processing capacity in the cerebral cortex by disrupting synaptic homeostasis and triggers neuroinflammatory processes, leading to excessive excitability in central auditory pathways [40, 42]. At the psychological level, insufficient sleep increases anxiety by weakening emotional regulation, resulting in increased sensitivity to sounds and decreased tolerance [43, 44]. Although our findings strongly support the vicious cycle between sleep disorders and sound tolerance, reliance on self-report measures represents a limitation. Therefore, future studies employing neuroimaging techniques to directly investigate the neuroinflammatory and psychological mediating mechanisms will be crucial for more comprehensively elucidating the pathophysiology underlying these interactions.

Psychological factors such as anxiety, stress, and depression may affect sleep quality, and Bayoumy et al. [45] also found significant associations between these factors and sleep quality. Many studies have shown that sleep disorders, such as sleep deprivation, which leads to poor sleep quality, are strongly associated with anxiety [18, 46]. Vidović et al. [47] found a moderate positive correlation between total PSQI scores and anxiety and reported that anxiety is a significant predictor of poor sleep quality. In the present study, higher Beck Anxiety scores were significantly associated with higher PSQI scores, suggesting that elevated anxiety levels may be linked to poorer sleep quality. Additionally, Beck anxiety scores were found to negatively impact SSQ subscales while increasing hyperacusis, phonophobia, and misophonia scores. This suggests that anxiety has negative effects not only on sleep quality but also on sound tolerance and auditory skills in daily life. Given the negative effects of high anxiety levels, interventions aimed at reducing anxiety levels may be beneficial in mitigating these negative effects. Therefore, a multidisciplinary approach is required.

### Limitations

This study examined the multidimensional effects of sleep quality by investigating the impact of sleep quality on auditory performance, sound tolerance, and anxiety levels in young adults based on subjective measurements. While the lack of objective sleep assessments (such as polysomnography) and auditory tests is a limitation, the aim of this study was to investigate how sleep quality is experienced in daily life and its impact on self-reported auditory performance, sound tolerance, and anxiety. This allowed us to access a larger sample group, and the findings provided valuable information about individuals’ real-life perceptions and individual differences. The strengths of this study include its relatively large sample size and its examination of sleep quality effects across multiple dimensions rather than focusing on a single aspect. Additionally, age homogeneity was achieved by selecting a study sample between the ages of 18 and 30, allowing for control of potential age effects. Although gender distribution was unbalanced, gender was not a statistically significant predictor in multivariate analyses, allowing for meaningful and reliable assessment of overall effects by focusing on a large sample of young adults. However, this study has several limitations that should not be overlooked. The cross-sectional design of the study precludes any causal inference due to the inability to determine the temporal direction of the observed relationships. The use of self-reported measures may have introduced recall bias and subjective reporting errors, potentially impacting the accuracy of the findings. Additionally, unmeasured confounding factors such as objective hearing thresholds, alcohol consumption, stress, and socioeconomic status may have influenced the observed associations, further supported by the relatively low R² values ​​in our regression models. Therefore, future longitudinal studies that include different age groups and balanced gender distribution, use objective assessments, and control for potential confounding variables are recommended to clarify causal relationships.

## Conclusion

This study, based on self-report measures, demonstrated significant associations between sleep quality and auditory performance in daily life, reduced sound tolerance, and anxiety levels in young adults with poor sleep quality. Decreased sleep quality was found to be associated with lower reported auditory performance and sound tolerance, and was also observed to be associated with higher anxiety levels. These findings, although based on subjective assessments, highlight the effects of sleep on various systems and emphasize its physiological necessity for healthy functioning. Considering the multidimensional effects of sleep quality, it is believed that adopting multidisciplinary approaches involving audiology, psychology, and neuroscience disciplines will enable a comprehensive assessment of sleep disorders and significantly contribute to guiding the development of rehabilitation practices in clinical applications.
